# Mistreatment of women in public health facilities of Ethiopia

**DOI:** 10.1186/s12978-019-0781-y

**Published:** 2019-08-27

**Authors:** Ephrem D. Sheferaw, Young-Mi Kim, Thomas van den Akker, Jelle Stekelenburg

**Affiliations:** 1 Jhpiego Ethiopia, Addis Ababa, Ethiopia; 2 Department of Health Sciences, University of Groningen, University Medical Centre Groningen, Global Health, Antonius Deusinglaan 1, 9713 AV Groningen, The Netherlands; 3 Jhpiego, Baltimore, USA; 4 Department of Obstetrics and Gynecology, Leiden University Medical Center, Leiden, The Netherlands; 5 Department of Obstetrics and Gynecology, Leeuwarden Medical Center, Henri Dunantweg 2, 8934 AD Leeuwarden, The Netherlands

**Keywords:** Mistreatment, Disrespect and abuse, Respectful maternity care, Ethiopia

## Abstract

**Background:**

Recent evidence suggests that mistreatment of women during childbirth is a global challenge facing health care systems. This study seeks to explore the prevalence of mistreatment of women in public health facilities of Ethiopia, and identify associated factors**.**

**Methods:**

A two-stage cross sectional sampling design was used to select institutions and women. The study was conducted in hospitals and health centers across four Ethiopian regions. Quantitative data were collected from postpartum women. Mistreatment was measured using four domains: (1) physical abuse, (2) verbal abuse, (3) failure to meet professional standards of care, and (4) poor rapport between women and providers. Percentages of mistreatment and odds ratios for the association between its presence and institutional and socio demographic characteristics of women were calculated using bivariate and multivariable logistic regression modeling.

**Results:**

A total of 379 women were interviewed, of whom 281 (74%) reported any mistreatment. Physical and verbal abuse were reported by 7 (2%) and 31 (8%) women interviewed respectively. Failure to meet professional standards of care and poor rapport between women and providers were reported by 111 (29%) and 274 (72%) women interviewed respectively.

Multivariable logistic regression analysis revealed that the odds of reporting mistreatment were higher among women with four or more previous births (aOR = 3.36 95%CI 1.22,9.23, *p* = 0.019) compared to women with no previous childbirth, Muslim women (aOR = 3.30 95%CI 1.4,7.77, *p* = 0.006) and women interviewed in facilities with less than 17 births per MNH staff in a month (aOR = 3.63 95%CI 1.9,6.93, *p* < 0.001). However, the odds of reporting mistreatment were lower among women aged 35 and older (aOR = 0.22 95%CI 0.06, 0.73, *p* = 0.014) and among women interviewed between 8 and 42 days after childbirth (aOR = 0.37 95%CI 0.15, 0.9, *p* = 0.028).

**Conclusion:**

Mistreatment during childbirth in Ethiopia is commonly reported. Health workers need to consider provision of individualized care for women and monitor their experiences in order to adjust quality of their services.

**Electronic supplementary material:**

The online version of this article (10.1186/s12978-019-0781-y) contains supplementary material, which is available to authorized users.

## Plain English summary

Recent evidence suggests that mistreatment of women during childbirth is a global challenge facing health care systems. This study seeks to explore the level of mistreatment of women in public health facilities in Ethiopia and identify associated factors**.** The study was conducted in hospitals and health centers across four Ethiopian regions. Quantitative data were collected from postpartum women. Mistreatment was measured using four domains: (1) physical abuse, (2) verbal abuse, (3) failure to meet professional standards of care, and (4) poor rapport between women and providers. A total of 379 women were interviewed, of whom 281 (74%) reported any mistreatment. Physical and verbal abuse were reported by 7 (2%) and 31 (8%) women interviewed respectively. Failure to meet professional standards of care and poor rapport between women and providers were reported by 111 (29%) and 274 (72%) women interviewed respectively. The odds of reporting mistreatment were higher among women aged less than 25 compared to women aged 35 and above, those with four or more previous births compared to no previous birth, and also in those who gave birth in facilities with fewer (less than 17) births per MNH staff in a month. Mistreatment during childbirth in Ethiopia is commonly reported. Health workers need to consider provision of individualized care for women and monitor their experience in order to adjust quality of their services.

## Introduction

The third Sustainable Development Goal aims to reduce the maternal mortality ratio (MMR) to below 70 per 100,000 live births in all countries by 2030 [1]. Ensuring access to skilled birth attendance in well-functioning health facilities is a widely-accepted strategy to prevent maternal mortality [2] . Recent studies in low- and middle-income countries on experiences of women during childbirth in health facilities have revealed unacceptable practices including disrespectful, abusive or neglectful treatment [3–6]. These experiences of mistreatment are identified as reasons for low institutional birth rates [7–10].

Ethiopia saw a dramatic decline in MMR from 1400 to 420 per 100,000 live births between 1990 and 2013 [11]. Despite this progress, the MMR remains unacceptably high. Ensuring access to maternity care by skilled providers working in a functional health facility forms the basis of the strategy formulated by the Federal Ministry of Health of Ethiopia to reduce maternal mortality. As part of this strategy, a large number of health centers and hospitals were built and staffed by essential health care providers over the past decade. Coverage of births attended in health facilities increased from 7 to 62% between 2007 and 2015 [12, 13]. The Ethiopian health system is structured into three tiers: primary, secondary and tertiary levels. The primary care level includes primary hospitals, health centers and health posts. The secondary level includes general hospitals and the tertiary level comprises specialized hospitals [14]. Most of the expansion in the health sector over the past decade occurred at the primary level [15, 16].

Although the Ministry of Health promotes the provision of compassionate and respectful care in these facilities, which includes individualized and culturally sensitive care for all women [17], some studies in Ethiopia indicate that physical and verbal abuse, non-consented care and lack of consideration of cultural practices related to childbirth by health workers may take place, possibly compounded by the increasing pressure on the health system due to the growing number of facility births [10, 18]. In this way, disrespectful and abusive behaviors by health providers during childbirth, which are known to be a significant barrier to increasing facility based births, could be a threat to the gains made in coverage of skilled birth attendance and to reductions of maternal mortality [19, 20].

Understanding the prevalence of mistreatment in Ethiopian maternity care facilities is therefore critical. Studies to date are limited in number, conducted in a limited geographic area or fail to apply similar definitions. In a previous study in a hospital and two health centers in Addis Ababa, 78% of respondents experienced one or more categories of disrespect and abuse including violation of the right to information, informed consent, and choice of position during childbirth [21] A study in four health centers in Amhara and SNNP regions, 21.1% of women reported occurrence of any disrespect and abuse [22]. A study using provider-client observations in 28 facilities across the four most populous regions in Ethiopia showed that 36% of women experienced any mistreatment [23]. On the other hand, a community based assessment in Tigray region reported that 22% of women experience mistreatment during childbirth in health facilities [24].

This study aimed to generate evidence on the prevalence of mistreatment of women in public health facilities as reported by women in Ethiopia and identify factors that may contribute to such mistreatment.

## Materials and methods

### Design

The study used a cross-sectional two-stage sampling design with quantitative data collection methods.

### Setting

The study was conducted in June 2016, in 38 public hospitals and health centers across 4 regions in Ethiopia- Oromia, Amhara, Southern Nations Nationalities and Peoples (SNNP) and Tigray. Interviews were conducted in public hospitals and health centers in both urban and rural areas.

### Data collection

Twelve data collectors, who were external to and clearly expressed not to be part of facility staff with a minimum of a BSc degree qualification, conducted the recruitment in postnatal and immunization units. Data collectors interviewed women in a private area within the premises of the health facilities immediately after childbirth or after women attended immunization and postnatal care services. Quantitative data on health facility policy were collected from facility managers and maternity unit leaders.

Four supervisors and two coordinators from the Maternal and Child Survival Program (MCSP) and the Ministry of Health coordinated the data collection process. Data collectors were external to the health facilities assessed. Study coordinators ensured that data collectors were competent in the application of the standardized tools for data collection. All data collectors attended a three-day training workshop in Addis Ababa to ensure that they were oriented to scientific and ethical standards.

### Participants

Maternity unit leads were interviewed about facility-related policies such as allowing non-harmful cultural practices during childbirth in health facilities and allowing women to choose their preferred birthing position. Women who had used skilled birth attendance services in public health facilities from 6 hours to 3 months prior to the start of data collection were included and interviewed about their birthing experiences.

### Data sources

Since no validated tool for measuring mistreatment of women at the time of data collection was present in the literature, the study team utilized a structured interview tool for postpartum women adopted from the Population Council Heshima project that was piloted in Kenya and previously applied in Kenya, Tanzania and Ethiopia [16, 25]. The exit interview tool captured four of the seven types of mistreatment. These are: 1. Physical abuse, 2. Verbal abuse, 3. Failure to meet professional standard of care and 4. Poor rapport between women and providers [26]. For facility-related policy assessment, a survey tool was developed by the study team. The tools used are included in Additional files 1 and 2.

The outcome variable was any mistreatment, measured as a binary (yes/no) variable, which was defined as being present if any of the four categories of mistreatment was reported. Physical abuse included hitting, slapping or pinching. Verbal abuse included shouting, scolding, threatening to take women into the operating theatre or addressing women using insulting names. Failure to meet standards of care included neglecting women when they needed care at some point during labor and childbirth, ignoring women’s requests for pain relief, providing treatment without consent and providing care that violated privacy of women. Poor rapport between women and providers included not greeting women, not explaining the labor progress, not responding to women’s questions in a polite manner, not encouraging women to move around freely, not allowing women to bring a companion, not allowing women to give birth in their preferred birth position and not offering hot drinks or food after childbirth. Based on literature review and expert judgment of the investigators the following explanatory variables were assessed: socio demographic characteristics of women including age, educational status, marital status, employment status, number of previous births, religion, residence, antenatal care, follow-up visit, time of childbirth and interval between time of interview and childbirth. Similarly, facility-related explanatory variables such as facility type, a policy of organizing facility visits for pregnant women, a policy of reporting providers’ misconduct, number of births per maternity care worker, and the proportion of maternity care providers trained in BEmONC were assessed.

### Sampling

The sample size calculation for the client interview used assumptions of 95% level of confidence, variability of attributes related to Disrespect and Abuse (D&A) with a proportion of 0.14 (using the MCHIP study estimate of self-reported D&A prevalence in the same regions in 2014 [27]), and an anticipated non-response rate of 10%, plus or minus 4 percentage points of relative error (which is equivalent to 0.56% absolute margin of error), 10% non-response rate and using Design Effect (DE) of 1.2 since there were no estimates of DE from previous studies [28]. Using these statistical parameters, the total number of participants required for client interviews was 382. However, we planned to interview 380 women by allocating an equal number of ten clients from each facility using the strategy described below.

Sampling was conducted in two stages. First, 85 hospitals and 751 health centers that have an average of 60 births per month from the national health management information systems report were listed as sampling frame. These facilities were categorized into two groups, high volume and low volume facilities, using the median number of attended births per month; using power allocation 11 hospitals and 27 health centers (19 from high volume and 19 from low volume) were selected randomly using a systematic random sampling approach. In the second stage of sampling, 10 women from each selected facility were selected randomly in postnatal and immunization units. All clients that fulfilled inclusion criteria, having attended childbirth services in selected health facilities between 6 hours to 3 months prior to the interview were invited.

### Data analysis

The study team leader supervised data entry and cleaning. Data were entered using EPI data software and exported to Stata 15.0 for further statistical analysis [29]. Before data analysis was started, the presence of extreme values was assessed using standardized scores of independent variables. Similarly, the effect of influential cases and leverage cases were assessed using residual analysis.

Frequencies and percentages of client background characteristics and birth experiences, availability of facility policies related to respectful maternity care (RMC) and components of mistreatment disaggregated by health center and hospital were calculated. Bivariate analysis was performed to detect statistically significant associations between the outcome variable (mistreatment of women) and explanatory variables in the study group. Multivariable multilevel logistic regression analysis was used to identify factors associated with mistreatment of women. A *P*-value less than 0.25 in the binary analysis was used as the criterion to include a variable into the multivariable regression model. The explanatory variables included in the binary and multivariable regression were women’s individual characteristics (age, education level, religion, marital status, parity, residence, time of birth and presence of complications at birth). Health facility characteristics recorded were proportion of maternal and child health care (MCH) providers trained in Basic Emergency Maternal Obstetric and Newborn Care (BEmONC), number of birth per MCH provider, availability of a policy of providing a tour for pregnant women around the maternity unit and availability of a policy of anonymous reporting of providers’ misconduct. The effect sizes of individual and facility level factors on the reported mistreatment of women were expressed in crude odds ratios (OR) and adjusted odds ratios (aOR), with their respective 95% Confidence Intervals (CI).

### Ethical considerations

This study was approved by the Johns Hopkins Bloomberg School of Public Health Institutional Review Board in Baltimore, Maryland, USA. The institutional review board ruled the protocol exempt from review under 45 CFR 46.101(b)(5). The study was further approved by the national Ministry of Health and the regional health bureaus of Amhara, Oromia, Tigray and SNNPR. Structured interviews of women were conducted in a private area after receiving oral informed consent. The client consent forms were translated into and administered in the Amharic, Tigrigna and Afan Oromo languages.

## Results

A total of 379 women were interviewed in 27 health centers and 11 hospitals in Oromia, Amhara, Tigray and SNNPR regions. Among 380 women we planned to interview, we could not interview three women in one of the hospitals because of temporary civil unrest in the town and an additional two women were interviewed in two other health centers. 

A majority, 73 of the 107 (68%) participants interviewed in hospitals were urban residents compared to only 121 of the 272 (44%) participants interviewed in health centers. The percentage of women interviewed in the first week after childbirth was higher in health centers compared to hospitals (41% vs. 11%) (Table 1).

**Table 1:** Background characteristics and birth experience of respondents

Variables	Total (*N* = 379)		Health Centers (*N* = 272)		Hospitals (*N* = 107)	
	N	Percent	N	Percent	N	Percent
Residence Location						
Urban	194	51	121	44	73	68
Rural	185	49	151	56	34	32
Residence region						
Tigray	40	11	20	7	20	19
Amhara	100	26	70	26	30	28
Oromia	139	37	112	41	27	25
SNNPR	100	26	70	26	30	28
Age						
< 25	167	44	116	43	51	48
25–34	177	47	129	48	48	45
35+	34	9	26	10	8	7
Education - ever attended school	278	73	192	71	86	80
Highest level of school attended						
Informal education/can read and write	7	3	6	3	1	1
Primary (1–8)	137	50	106	56	31	36
Secondary	86	31	51	27	35	41
TVET/College/University	46	17	27	14	19	22
Religion						
Muslim	107	28	87	32	20	19
Orthodox Christian	195	52	130	48	65	61
Other Christian (protestant, catholic etc)	76	20	55	20	21	20
Marital status						
Never married / Single/divorced	12	3	8	3	4	4
Currently married or co-habiting	366	97	263	97	103	96
Employment status						
Not employed /house wife	264	70	199	73	65	61
Employed	97	26	61	23	36	34
Student	17	4	11	4	6	6
Parity - births ever had including most recent						
1	162	43	104	38	58	54
2–3	115	30	85	31	30	28
4 +	102	27	83	31	19	18
Duration between birth and interview						
Less than a week	73	19	29	41	44	11
1–6 weeks	136	36	99	35	37	36
7–12 weeks	170	45	144	24	26	53
Time of birth						
Night time	179	47	131	45	48	48
Day time (6:00 AM-6:00 PM)	199	53	140	55	59	52

Table 2 describes policies on RMC in the facilities. Health facility managers and maternity unit leaders reported existence of most of the policies on RMC. The least reported policies were allowing non-harmful cultural rituals in health facilities (reported in 23 (85%) health centers and 4 (36%) hospitals) and allowing women a choice of birthing position (in 20 (74%) health centers and 6 (55%) hospitals).

**Table 2:** Availability of Facility based Policies on Respectful Maternity Care, *N* = 38

Availability of Policy related to RMC	Total (*n* = 38)		Health Center (*n* = 27)		Hospital (*n* = 11)	
	N	%	N	%	N	%
Freedom of movement during labor (i.e., walking around)	38	100	27	100	11	100
Prevention of institutional violence against women and newborns	38	100	26	100	12	100
Requirement of informed consent for procedures	36	95	25	93	11	100
Keeping the newborn with the mother immediately after the birth	34	89	25	93	9	82
Admission of family members/ person of choice to accompany women during labor/childbirth	33	87	24	89	9	82
Keeping mother and baby together in the facility	34	89	25	93	9	82
Policy of allowing non harmful cultural rituals in the facility	27	71	23	85	4	36
Allowing women a choice of position for birth	26	68	20	74	6	55

**Table 3:** Types of mistreatment reported by women, *N* = 379

Categories	Total		Health center		Hospitals	
	N	%	N	%	N	%
Any mistreatment	281	74	188	69	93	87
Physical abuse: hit /slapped/pinched by the provider**	7	2	5	2	2	2
Verbal abuse: shouted at, scolded, threatened with going to operating theatre, called by insulting name	31	8	20	7	11	10
Failure to meet professional standards of care: (at least one of the 4)	111	29	80	29	31	29
Neglect: Client left unattended when needed care at any point in stay	39	10	28	10	11	10
Client’s request for pain medication was ignored: (among those that requested it; *N* = 117, Health Center = 80, Hospital =37)	43	37	36	45	7	19
Non-consented care: Any treatment done without women’s permission**	59	16	38	14	21	20
Non-confidential care: At any point during Labor and childbirth stay client were treated in a way that violated privacy	24	6.3	18	6.6	6	5.6
Poor Rapport between women and providers: (at least one of the 7)	274	72	181	67	93	87
Poor Reception: The health workers did not greet woman when she came to this facility during Labor and childbirth	64	17	41	15	23	21
No Explanation during labor: The health workers did not explain the next steps during Labor and childbirth to clients	181	48	154	43	44	41
Not Responding to questions: The health workers did not respond to clients’ questions politely	212	16	24	15	14	18
Free Movement not encouraged: Health workers do not encourage women to walk and change positions during Labor and childbirth	113	30	73	73	40	63
Not Allowing Birth companion during labor	105	28	69	25	36	34
Birth Position of women choice was not respected: The health workers did not allow women to give birth in the position they wanted during Labor and childbirth	217	56	142	51	75	69
Food and drink not offered: After childbirth, women were not offered hot drinks or food	62	16	39	14	23	21

*• P*-values are from logistic regression ** three missing cases

**Table 4:** Logistic regression analysis of socio-demographic variables of women and Environmental characteristics on the reported mistreatment of women

	Bi-variable			Multivariable		
Mistreatment	OR	[95% CI]	P-value	aOR	[95% CI]	P-value
Age category (ref: < 25)						
25–34	1.51	0.73,3.11	0.266	1.00	0.47,2.13	0.999
35+	0.68	0.23,2.04	0.491	0.22	0.06,0.73	0.014*
Marital status (ref: Currently married)						
Single or divorced	1.75	0.18,17.5	0.633			
Parity (Ref. 1)						
2 to 3	0.62	0.28,1.37	0.239	0.94	0.44,1.98	0.867
4+	1.80	0.75,4.33	0.189	3.36	1.22,9.23	0.019*
Education level (ref: TVET/College/University.)						
informal/no education	1.96	0.05,81.71	0.724			
Primary (1–8)	1.72	0.41,7.26	0.462			
Secondary	0.66	0.16,2.67	0.557			
Religion (ref: Orthodox Christian)						
Muslim	3.24	0.95,11.13	0.061	3.30	1.4,7.77	0.006*
other Christians (Prot., catholic)	1.39	0.32,6.11	0.662	1.61	0.63,4.09	0.317
Facility (ref: Health center)						
Hospitals	9.63	1.25,74.26	0.03	2.09	0.92,4.76	0.077
Region						
igray	2.04	0.11,36.56	0.63	1.21	0.35,4.2	0.759
Amhara	0.88	0.12,6.65	0.903	1.67	0.7,4.01	0.248
SNNPR	0.09	0.01,0.7	0.021	0.19	0.08,0.44	< 0.001*
Residence (ref: rural)						
Urban	1.26	0.52,3.04	0.607			
ANC visits (ref. < 4)						
4+	1.69	0.78,3.67	0.183	1.26	0.67,2.38	0.47
Time from childbirth to interview (ref: First week)						
1–6 weeks	0.37	0.12,1.12	0.079	0.37	0.15,0.9	0.028*
7–12 weeks	0.66	0.21,2.04	0.470	0.88	0.35,2.21	0.779
Childbirth time (ref. night)						
Day time	1.31	0.66,2.59	0.434			
Monthly childbirth per MNH Staff (Ref. > 17)						
Less than 17	5.46	0.97,30.62	0.054	3.63	1.9,6.93	< 0.001*
MNH staff trained in BEmONC (Ref. < 50%)						
50% or more	3.41	0.62,18.69	0.157	1.45	0.75,2.82	0.27
Policy of facility tour for pregnant women (ref. yes)						
No	6.74	1.23,37.02	0.028	1.72	0.89,3.32	0.11
Policy of anonymous (ref. yes)						
No	3.27	0.2,52.57	0.402			

* Ref: Reference group. aOR: Adjusted odds ratio, * Statistically significant at Alpha = 0.05

Table 3 describes the level of self-reported mistreatment of women. Overall, three out of four women (74%, *n* = 281) reported any mistreatment during their latest childbirth experience in health facilities, with women in hospitals and health centers reporting 87 and 69% respectively.).

Physical abuse and verbal abuse were the least prevalent experiences of mistreatment reported by seven (2%) and 31 (8%) women respectively. Failure to meet standards of care (neglect, non-consented care, non-confidential care and pain relief ignored) was reported by 29%. On the other hand, poor rapport between women and providers was the most prevalent form of mistreatment, reported by 72% of the women. Standardized scores of independent variables confirmed that there were no extreme values. Similarly, residual analysis suggested the absence of influential and leverage cases.

Table 4 describes bivariate and multivariable logistic regression analysis of possible predictors of mistreatment of women.

In the bivariate analysis, compared to women interviewed in health centers those interviewed in hospitals (OR = 9.63 95%CI 1.25, 74.26, *p* = 0.03) were more likely to report mistreatment. Women who gave birth in health facilities with less than 17 births per month (OR = 5.46, 0.97, 30.62, *p* = 0.054) and with no policy of a facility tour for pregnant women (OR = 6.74 95% CI 1.23, 37.02; *p* = 0.028) were more likely to report mistreatment.

In a multivariable logistic regression analysis, the odds of reporting mistreatment were higher among women with four or more previous births (aOR = 3.36 95%CI 1.22, 9.23, *p* = 0.019) compared to women with no previous childbirth, among Muslim women (aOR = 3.30 95%CI 1.4, 7.77, *p* = 0.006) compared to Orthodox Christians and among women interviewed in facilities with less than 17 births per MNH staff in a month (aOR = 3.63 95%CI 1.9, 6.93, *p* < 0.001). However, the odds of reporting mistreatment were lower among women aged 35 and older compared to those younger than 25 (aOR = 0.22 95%CI 0.06, 0.73 *p* = 0.014) and among women interviewed between 8 and 42 days after childbirth (aOR = 0.37 95%CI 0.15, 0.9 *p* = 0.028).

## Discussion

This study assessed the level of mistreatment of women during childbirth in 38 randomly selected health facilities with high and low case load across Tigray, Amhara, Oromia and SNNP regions in Ethiopia. The four regions included in the study represent more than 86% of the total population of the country [30]. Policies of RMC at facility level that aim to improve women’s experiences, including allowing a birth companion of choice, keeping newborn and mother together following childbirth, and allowing women to take their preferred birthing position were not universally observed. The observed discrepancy could be due to lack of focus by the leadership of health facilities and lack of monitoring on the policies by district and regional level health managers. A systematic review on RMC policies previously showed that such policies are feasible in low resource settings if these are prioritized [31].

Three-fourth of women interviewed reported experiencing any mistreatment. These findings are consistent with previous studies from the Ethiopian cities Addis Ababa and Bahirdar in which 78 and 67.1% of women reported disrespect and abuse respectively [21, 32]. However, our finding was higher than three other studies conducted in Ethiopia reporting 21–36% of mistreatment based on provider-client structured observation [23, 24, 33].

Physical abuse was reported by only 2 % women which was comparable to a study in Addis Ababa that reported 2.3% physical abuse [21] and a study in Tanzania that reported 2.7% physical abuse in exit interview [5]. Our finding was higher than a study conducted in Tigray region that reported 0.8% physical abuse [24] and a study conducted in Amhara and SNNPR regions that reported 0.5% physical abuse [33]. Our finding was lower than a previous study in the same four regions in Ethiopia that reported 9% physical abuse using structured observation [23] and much lower than a community based study among women in Bahirdar town that reported 23.2% physical abuse [32].

Verbal abuse was reported by 8 % women which was comparable to our previous study in the same four regions in Ethiopia that reported 8 % verbal abuse [23], a study in Addis Ababa that reported 7.5% insult, intermediation, threat or coercion [21] and a study in Tanzania that reported 8.7% women being shouted at [34]. But the reported level of verbal abuse was lower than a study in Bahirdar town that reported 27.1% of women reported verbal insult committed by providers [32] and a study conducted in Tigray region that reported 12.5% women being shouted at and 10.5% women being scolded [24].

Nearly one in three women reported failure to meet professional standards of care that included being left unattended (10%), pain relief medication being denied (37%), non-consented care (16%) or non-confidential care (6.3%). Previous studies in Ethiopia did not use a comprehensive definition for failure to meet professional standard of care but different studies reported its components. The finding reported for components of failure to meet professional standards of care was consistent with a study conducted in Amhara and Oromia regions that reported 15.2% women experienced violation of privacy, 17.8% women experienced non consented care [22] and a study conducted in Tanzania that reported 8.7% women were left unattended [5]. The finding on some components were not consistent with our previous study in the four regions that reported 17% women experienced violation of privacy and 19% women experienced being left unattended [23] and a study in Tigray region that reported 6% women were left unattended [24].

Nearly three out of four women experienced poor rapport with providers that include poor reception of women (17%), next steps not explained (48%), not responding to women questions (16%), not allowing birth companion (28%) and not allowing women preferred birth position (56%). The findings on poor rapport between women and providers was one of the first finding to our knowledge. Other studies assessed components of poor rapport between women and providers. The reported level of poor reception was lower than previous study in the same regions that reported 23% women were not greeted and received politely [23]. The reported level of poor communication was higher than previous study that reported 35% women did not receive explanation about next steps [23].

Our findings show that women younger than 25 years were more likely to report mistreatment compared to those 35 years and above. This finding for younger groups of women is consistent with other studies in South Africa, Uganda and rural Australia suggesting that young women may be more likely to be mistreated or discriminated against by health providers, and sometimes blamed for getting pregnant at a younger age [26, 35–38]. It is possible however, that compared to the older age group, these women have different expectations from the health system and/or have been sensitized to respectful care to a larger extent. Older women may either have normalized the experience of mistreatment [6] or may feel barriers to report it. Muslim women were more likely to report mistreatment compared to Orthodox Christians. It is unclear whether cultural and religious expectations of Muslim women related to privacy and sex of the care provider play a role. Alternatively, Muslim women could be discriminated against by care providers. A study in Afar, Ethiopia, a predominantly Muslim community, revealed that women did not seek maternity care from health facilities because of poor services and unfriendly or even abusive treatment during childbirth [39]. A study in Ghana suggested that Muslim women did not seek maternity care from health facilities because health care providers’ lack of knowledge and insensitivity to religious and cultural practices of Muslim women [40].

Women interviewed during eighth to 42 days after childbirth were less likely to report mistreatment compared to those interviewed in the first 7 days. The reason for reporting higher rates of mistreatment during the first 7 days after childbirth and lower rates after the seventh day could be due to a fresh memory of the birthing experience in the first week. Women may not have reported negative experiences for fear of reprisal by health care providers during their visit for immunization. A previous study in Tanzania in which women were interviewed in health facilities after childbirth and after a 5–10 week follow-up showed an increase in the level of mistreatment reported, considering that the follow-up interview was held at the woman’s home [5].

Women with four or more previous births were more likely to report mistreatment. Discrimination of women based on parity was identified in a systematic review [26]. This finding is consistent with a study in Kenya that reported women with four to nine previous births being more likely to experience some form of mistreatment including non-consented care, detainment for lack of payment and being requested for bribes [25]. Facility level factors significantly associated with mistreatment of women were the number of births per MNH provider. Women interviewed in facilities with lower numbers of monthly births (< 17) per MCH provider were more likely to report mistreatment. With increasing numbers of births per MNH provider, the odds of reporting mistreatment decreased. This is consistent with the finding of a systematic review conducted in five African countries, which indicated that facilities with a low case load were associated with poor quality of basic maternity care services [41].

The reason for lower levels of mistreatment in facilities with relatively high numbers of births per MCH provider could have a causal relationship, and the other way around. In other words, high or low volume of clients could be the result of previous treatment that women experienced in these facilities, either attracting them to come or making them give birth elsewhere. However, this finding contradicts with assumptions suggesting that health providers mistreated women due to high work load. High workload was identified as a cause of negative attitudes and behavior of maternity care providers in a systematic review in low- and middle-income countries [42]. Another reason for the increase in mistreatment in facilities with lower maternity case load per provider could be the rapid expansion of these facilities since most of the new facilities usually have low case load due to preference of women for previously established facilities.

This study measured prevalence of mistreatment, using a nationally representative sample of health facilities in the four largest regions of the country. However, there are some limitations. This assessment was conducted on the premises of health facilities instead of women’s homes. This may create courtesy bias, i.e. women may have provided socially desirable responses to data collectors because of fear of repercussions during postnatal care visits. To mitigate this problem, data collectors were trained to ensure privacy and confidentiality of information. Another limitation could be recall bias leading to underreporting of some of the events, since interviews were conducted within the same day to 3 months after childbirth. Those women interviewed weeks after childbirth may have forgotten some of the interactions with health providers that would have been categorized as mistreatment. However, earlier studies on birthing experiences of women reported that women remember negative experiences for long periods of time [43]. Another limitation, inherent to study design, is the fact that residual confounding variables such as unmeasured provider and facility characteristics may have affected study findings.

## Conclusions

This study identified that most women experienced some form of mistreatment during childbirth in Ethiopian health facilities. Younger women, women with four or higher previous childbirths, Muslim women, women who received childbirth services in health facilities with low numbers of births per provider were disproportionately affected by mistreatment. Health workers efforts to improve respectful maternity care should consider such factors that are associated with mistreatment of women in health facilities. Health providers need to provide culturally sensitive women-centered care considering particular needs of each woman (younger versus older women) and continuously monitor experiences of women. National and regional level policy makers and program managers should investigate reasons for lower case load per provider in some health facilities so as to make appropriate corrective measures.

## Additional files

Additional file 1: Exit interview tool. (PDF 589 kb)

Additional file 2: RMC policy assessment tool. (PDF 151 kb)

## Data Availability

The datasets used during the current study is available from the corresponding author on reasonable request.

## Abbreviations

aOR: Adjusted Odds Ratio
BEmONC: Basic Emergency Obstetrics and Newborn Care
CI: Confidence Interval
D&A: Disrespect and Abuse
DE: Design Effect
MCSP: Maternal and Child Survival Program
MMR: Maternal Mortality Ratio
MNH: Maternal and Newborn Health
MOH: Ministry of Health
OR: Odds Ratio
RMC: Respectful Maternity Care
SNNPR: Southern Nation Nationalities Peoples Region
USAID: United States Agency for International Development
