# Supplementary material for: Mistreatment of women in public health facilities of Ethiopia
Source: Reprod Health. 2019 Aug 27;16:130. doi: 10.1186/s12978-019-0781-y (PMC6712647; doi:10.1186/s12978-019-0781-y)
Supplement: Supplementary file 1 — Exit interview tool. (PDF 589 kb) [file 12978_2019_781_MOESM1_ESM.pdf]

MCSP ETHIOPIA- MEASURING RESPECTFUL MATERNITY CARE (RMC)
AMONG POSTPARTUM AND CHILD IMMUNIZATION CLIENTS

መለያ ID: [___|___]___|___|___]___|___]

Tool A የተገልጋዮች ቃለ-መጠይቅ ማድረጊያ መጠይቅ/ Client Interview Questionnaire

Region	ትግራይ/ Tigray [_0__| 1___] አማራ/Amhara [__0_|_3__]<br>ኦሮሚያ/Oromiya [__0_|_4__] ደብብህክ/SNNPR [__0_|_7_]	[___|___]
Facility name:	___________	የጤና ተቋሙ በጥናቱ የተሰጠው ኮድ<br>Fac. Code for Study: (see fac. Code attached)<br>___ ___ ___ ___ ____
የጤና ተቋሙ ዓይነት/Facility type	01-ሪፈራል ሆስፒታል/ Referral Hospital<br>02-የመጀመሪያ ደረጃ ሆስፒታል/Primary Hospital<br>03-ጤና ጣቢያ/Health Centre	[___|___]
የቃለ-መጠይቁ ውጤቶች /Interview Outcomes	01 ተጠናቋል/Complete<br>02 በከፊል ተጠናቋል/ Partially completed<br>03 እንቢተኛ/ Refused<br>88=ሌላ (ይጥቀሱ)<br>Other(Specify)______________________	[___|___]

	ተቆጣጣሪ SUPERVISOR	ቃለ-መጠይቅ አድራጊ INTERVIEWER
Name	____________________	____________________
DATE (DD/MM/YY)	____________________	____________________

ቃለ-መጠይቅ የተደረገበት ሰአት፡- TIME INTERVIEW STARTED: [___|___:___|___]

የአገልግሎቱ/የተገልጋዩ ዓይነት የድህረ-ወሊድ ህክምና Service/Client type:
______a) የድህረ-ወሊድ ህክምና Postpartum/Postnatal Clinic
______b) የክትባቱ እና የጨቅላ ህጻናት ህክምና/Immunization (Child) Clinic
______c) የቤተሰብ ምጣኔ እና ድህረ ወሊድ ህክምና/ Family Planning Clinic – Postpartum Client

A.	የስነ-ህዝብ መረጃ Demographics	
	ስለእርስዎ እና ስለ ጤናዎ አንዳንድ ጥያቄ እጠይቅዎታለሁ፡፡<br>Now, I would like to ask you some questions about yourself and your health.	
	አብዛኛውን ጊዜ የት ነው የሚኖሩት? Where do you usually live?	1)ከተማ/ urban<br>2)ገጠር/rural
	እድሜዎ ስንት ነው? How old are you?	_________ ዓመት years (NUMERIC 15+)
	ት/ቤት ገብተው ያውቃሉ? Have you ever attended school?	1 አዎ Yes<br>0 የለም No → GOTO 5

	ከፍተኛ የደረሱበት የትምህርት ደረጃ ስንት ክፍል ነው?<br>What is the highest level of school you attended?	In formal education/can read and write<br>Primary<br>Secondary<br>TVET/College/University
	የምትከሉት ኃይማኖት ምንድን ነው?<br>What is your religion?	1 ሙስሊም Muslim<br>2. ኦርቶዶክስ ክርስቲያን Orthodox Christian<br>3. ሌላ ክርስቲያን፣ (ፕሮቴስታንት፣ ካቶሊክ ወዘተ)Other Christian (protestant, catholic etc)<br>96 ሌሎች (ይግለጹ) Other (specify) ______________________
	የጋብቻ ሁኔታዎ?<br>What is your marital status?	1 ያላገቡ Never married / Single<br>2 ጋብቻ የመሰረቱ ወይም ከትዳር ጋር አብረው የሚኖሩ Currently married or co-habiting<br>3 የተለያዩ/ተፋቱ Separated/ Divorced<br>4 የትዳር አጋር የሞተባቸዉ Widowed
	የሥራ ሁኔታዎ<br>What is your employment status?	ሥራ የማትሰራ/የቤት እመቤት Not employed /house wife<br>ሠራተኛ (የግል ሥራ/በሌላ አካላት ተቀጥረው የሚሰሩ)Employed (self-employed/employed by other entity)<br>ተማሪ Student
	በህይወት ዘመንዎ የአሁኑን ጨምሮ ስንት ጊዜ ወልደዋል?<br>Over the course of your life, how many births have you had including this one?	____________(NUMERIC (if 1 GOTO 10))
	ስንት ሴት ልጆች አሉዎት?<br>How many girl children do you have?<br>ስንት ወንድ ልጆች አሉዎት?<br>How many boy children do you have?	____ ሴቶች girls<br>____ ወንዶች boys
	ባለፉት 12 ወራት ውስጥ ለልጆችዎ ወይም ለራስዎ በተለያየ ምክንያት ስንት ጊዜ ወደ የህክምና ማዕከል ሄደሻል?<br>In the last 12 months, how many times have you been to a health care facility for your children or for yourself for any reason?	__________NUMERIC
	አሁን ለመጨረሻ ጊዜ ለነበርዎት ጽንስ ስንት ጊዜ የቅድመ ወሊድ አገልግሎት ክትትል በድምሩ አግኝተዋል?<br>How many antenatal (ANC) care visits did you make in total for your most recent pregnancy at health facility?	_____(numeric)
B	**Delivery Characteristics**	
	በመቀጠል አሁን ለመጨረሻ ጊዜ የነበርዎት የወሊድ አገልግሎት አንዳንድ ጥያቄ አነሳልዎታለሁ፡፡<br>Now I'm going to ask you some questions about your recent delivery	
12	መቼ ወለዱ? When did you give birth?	a).ከ __ ቀናት በፊት days ago OR<br>b). ከ__ ሳምንታት በፊት weeks ago
12A	በምን ጊዜ ወለዱ? What time did you give birth	1 ቀን Day time<br>0 ማታ Night time

13	በወሊድ ወይም በምጥ ወቅት ሌሎች ወገኖች በእርስዎ ምርጫ አብረዎት እንዲሆኑ ተፈቅዶሎታል?<br>Were you allowed to have the companion of your choice stay with you during labor?	1 አዎ Yes<br>0 የለም No
13A	በምጥ ወቅት ወይም በወሊድ ወቅት አገልግሎት ባገኙበት ቦታ ከእርስዎ ጋር የነበሩት ድጋፍ ሰጪ ሰዎች ከጎንዎት ነበሩ? Were you allowed to have the companion of your choice stay with you during child birth?	1 አዎ Yes<br>0 የለም No
14	በምጥ ወቅት ወይም በወሊድ ወቅት አገልግሎት ባገኙበት ቦታ ከእርስዎ ጋር የነበሩት ድጋፍ ሰጪ ሰዎች ከጎንዎት ነበሩ?<br>Did the companion stay with you during most of your labor, at your side?	1 አዎ Yes<br>0 የለም No
15	ምጥ በያዝዎት ወቅት አብርዎት የነበረው ማን ነው?<br>WHO was with you during labor?<br><br>የነበረው ሁሉ ላይ ምልክት ያድርጉ<br>CHECK ALL THAT APPLY	1. እናት Mother<br>2. ባል Husband<br>3. ሌላ የቤተሰብ አካል (የባል እናት፣ አባት፣ የባል አባት፣ እህት፣ የባል እህት፣ ሴት ልጅ፣ ወንድ ልጅ ወዘተ)<br>Other family members (Mother-in-law, Father, father-in-law, sister, sister-in-law, daughter, son etc)<br>4. ዘመድ ወይም ጓደኛ Relatives or Friends<br>5. የጤና ኤክስቴሽን ባለሙያዎች ወይም የማህበረሰብ በጎ ፈቃደኞች HEWs or community volunteers
16	ሰውየው ሴት ወይም ወንድ ነው? Was the person male or female	ወንድ Male<br>ሴትFemale<br>ሁለቱም ተፈቅዶላቸዋል Both were allowed
17	በወሊድ ጊዜ አብርዎት ማን ነበር?<br>WHO was with you during childbirth?<br>የነበረው ሁሉ ላይ ምልክት ያድርጉ፡፡<br>CHECK ALL THAT APPLY	1. እናት Mother<br>2. ባል Husband<br>3. ሌላ የቤተሰብ አካል (የባል እናት፣ አባት፣ የባል አባት፣ እህት፣ የባል እህት፣ ሴት ልጅ፣ ወንድ ልጅ ወዘተ) Other family members (Mother-in-law, Father, father-in-law, sister, sister-in-law, daughter, son etc)<br>4. ዘመድ ወይም ጓደኛ Relatives or Friends<br>5. የጤና ኤክስቴሽን ባለሙያዎች ወይም የማህበረሰብ በጎ ፈቃደኞች HEWs or community volunteers
18	በዚህ ወሊድ ዙሪያ ከዚያ በፊትም ሆነ በዚያን ወቅት ወይም ከዚያ በኋላ ወደዚህ ጤና ተቋም የመጡት ከሌላ ጤና ተቋም ሪፈር ተደርገው ነው?<br>Were you sent/referred from another facility to this facility at any point before, during, or after current delivery?	0 የለም ወደዚህ ጤና ተቋም የመጣሁት በቀጥታ ነው፡፡<br>No, I Came directly to this facility<br>1 በጤና ኤክስቴንሽን ባለሙያዎች ተልኬ ነው የመጣሁት<br>Sent from HEW<br>2 ከጤና ጣቢያ ተልኬ ነው የመጣሁት Sent from health center to this facility<br>3 ከሆስፒታል ተልኬ ነው የመጣሁት Sent from hospital to this facility

19	አሁን ከወሊድ ጋር የተያያዙ የጤና ቀውሶች እና የጎንዮሽ ምልክቶችን ልጠይቅዎት ነው፡፡ በወሊድ ጊዜ እና ከወሊድ በኋላ ወዲያው እነዚህን ምልክቶች ታይቶብዎታል? በመዘርዝሩ ላይ የቀረቡትን ምልክቶች ያንቧቸው (ይጠይቁ፡- እነዚህ ምልክቶች በወሊድ ወቅትም ሆነ ከወሊድ በኋላ ተከስተውብዎታል)Now I'd like to ask you about complications. Did you experience any of the following complications y, at the time of delivery of (NAME), or immediately after delivery?<br><br>READ EACH ITEM IN LIST. (Ask: Did this occur at the time of delivery or afterwards?)	ለእያንዳንዱ ምላሽ የተገለጋዩዋ እናት ምላሽ አዎ ከሆነ 1ን ቁጥር ያስቀምጡ፡፡ ምላሽዋ የለም ከሆነ 0ን ያስቀምጡ<br>For each response,<br>Write 1 if yes she had the complication<br>Write 0 if no she did not have the complication<br><br>ወሊድ Delivery ከወሊድ በኋላ<br>A. ራስ ምታት Headache……. ____ ____ _<br>B. ራስን መሳት/መንዘፍዘፍ Fits/convulsions ____ ___<br>C. የዕይታ መደብዘዝ ብዥታBlurry vision ____ ___<br>D. የደም ግፊት መጨመር High blood pressure __ ___<br>E. በብልት የደም መፍሰስ Vaginal bleedin ____ ___<br>F. ከፍተኛ ትኩሳት High fever….. ____ __<br>G. የተራዘመ ምጥ Prolonged labor…… ____ na<br>H. የሆድ ህመም Abdominal pain……. _____ ____<br>I. ሌላ (ይጥቀሱ)Other (specify) _______ __ ____
20	ከላይ ለተጠቀሱት የጎንዮሽ ምልክቶች ከጤና ተቋሙ ህክምና አግኝተዋል፡፡ Did you receive treatment at the facility for this complication?	1 አዎ Yes<br>0 የለም No
21a	የተሰጥዎት ህክምና ምንድን ነው?<br>What was the treatment?	_________________
21b	ህክምና ተሰጥትዎት ከሆነ ህክምናው ምን እንደሆነ ሰጪው ባለሙያ በግልጽ አብራርትዎልዎታል?<br>If you were given a treatment did the provider clearly explain what the treatment was?	1 አዎ Yes<br>0 የለም No
21c	ህክምናው ተሰጥትዎት ከሆነ ፈቃደኛ ስለመሆንዎ ተጠይቀዋል? If you were given a treatment were you asked for your permission or consent?	1 አዎ Yes<br>0 የለም No
	አሁን በጤና ተቋማት ላይ በተሰጥዎት የህክምና አገልግሎት ላይ ያለዎትን የእርካታ ደረጃ እጠይቅዎታለሁ፡፡ ማንኛውም የሚነግሩን ሚስጥር ለጤና ተቋሙ የማይጋለጥ ሲሆን የሚሰጡን ማንኛውም ምላሽ የእርስዎንም ሆነ የቤተሰብዎን አሁንም ሆነ በፊት የህክምና መገልገል መብት ላይ አሉታዊ ተጽዕኖ የለውም፡፡<br>I would like to ask you about how satisfied you were with your experience in this health facility. Please remember that nothing you tell us will be shared with the health facility, and your responses will not affect health care for you or your family in the future.	

MCSP ETHIOPIA- MEASURING RESPECTFUL MATERNITY CARE (RMC) AMONG POSTPARTUM AND CHILD IMMUNIZATION CLIENTS

C.	Perceived Quality and Satisfaction	
22	**አቀባበል፡-** በጽንስ እና ወሊድ አገልግሎት ወደ ጤና ተቋሙ በመጡ ጊዜ የጤና ተቋሙ ሠራተኛ ሠላምታ ሰጥቶ ተቀብልዎታል? Reception: Did the health worker receive and greet you when you came to this facility during L&D?	1 አዎ Yes<br>0 የለም No
23	**በአቀባበል/ምርመራ ወቅት የተደረገ ማብራሪያ፡-** በጤና ተቋሙ የጽንስ እና የወሊድ ህክምና አገልግሎት ለማግኘት በመጡ ጊዜ የምርመራውን እያንዳንዱን ደረጃ የጤና ባለሙያው በግልጽ አብራርተዎልዎታል?<br>**Explanation during admission/ examination:** During your L&D in this facility did the health workers explain each step of the examination to you?	1 አዎ Yes<br>0 የለም No
24	**በምጥ እና ወሊድ ወቅት የተደረገ ማብራሪያ፡-** በጤና ተቋሙ የጽንስ እና የወሊድ አገልግሎት ለማግኘት በመጡ ጊዜ በምጥ ወቅት ምን ሊከሰትልዎት እንደሚችል የጤና ባለሙያው አብራርተዎልዎታል?<br>**Explanation during labor:** During your L&D in this facility did the health workers explain what will happen in labour to you?	1 አዎ Yes<br>0 የለም No
25	**የጥያቄዎች ምላሽ፡-** በጤና ተቋሙ የጽንስ እና የወሊድ አገልግሎት ለማግኘት በመጡ ጊዜ የተቋሙ የጤና ባለሙያ ለጥያቄዎች ምላሽ በትህትና ሰጥተዎታል?<br>**Responding to questions:** During your L&D in this facility did the health workers respond to your questions politely?	1 አዎ Yes<br>0 የለም No<br>9 እናቶች አልጠየቁም NA (women did not ask)
26	**እንቅስቃሴ፡-** በጤና ተቋሙ የጽንስ እና የወሊድ አገልግሎት ለማግኘት በመጡ ጊዜ የተቋሙ ሠራተኛ በእግር እንዲራመዱ እና የአተኛነት አቅጣጫ መለዋወጥ እንዲችሉ የበኩሉን አስተዋጽኦ አድርጓል?<br>**Movement:** During your L&D in this facility did the health workers encourage you to walk and change positions	1 አዎ Yes<br>0 የለም No
27	**የወሊድ አተኛኘት አቅጣጫ፡-** በጤና ተቋሙ የጽንስ እና የወሊድ አገልግሎት ለማግኘት በመጡ ጊዜ የተቋሙ አዋላጅ በወለዱ ጊዜ እርስዎ በፈለጉት የወሊድ አቅጣጫ አዋልድዎታል?<br>**Position:** During your L&D in this facility did the health workers allowed you to give birth in the position you want	1 አዎ Yes<br>0 የለም No
28	**ምግብ፡-** ከወሊድ በኋላ ትኩስ መጠጥ ወይም ምግብ ተሰጥተዎታል? FOOD: After delivery, were you offered any hot drink or food?	1 አዎ Yes<br>0 የለም No
29	**እንክብካቤ፡-** በወሊድ ወቅት የተቋሙ ጤና ባለሙያዎች ያሳዩትን የእንክብካቤ እና ትህትና ደረጃ ምን ደረጃ ያወጡለታል?<br>**Caring:** How would you rate the caring and empathy (compassion) of the health workers at this facility for this delivery?	1 እጅግ በጣም ጥሩ Excellent<br>2 በጣም ጥሩ Very good<br>3 ጥሩ Good<br>4 በቂ Fair<br>5 ደካማ Poor
30	**አክብሮት፡-** በጤና ተቋሙ በዚህ ወሊድ ወቅት የጤና ተቋሙ ህክምና ባለሙያ ያሳየዎትን አክብሮት ምን ደረጃ ያወጡለታል? አክብሮት ማለቴ የጤና ባለሙያው የሰጥዎት እንክብካቤ እና እርስዎ በሚሰጡት መልክ በአክብሮት መንከባከቡን ነው፡፡<br>**Respect:** How would you rate the respect the providers showed you at this facility for this delivery? By respect I mean being treated with the care and attention you deserve.	1 እጅግ በጣም ጥሩ Excellent<br>2 በጣም ጥሩ Very good<br>3 ጥሩ Good<br>4 በቂ Fair<br>5 ደካማ Poor
31	በአጠቃላይ ሁሉንም ነገር ግምት ውስጥ በማስገባት በጤና ተቋሙ በዚህ ወሊድ ወቅት ያገኙትን እንክብካቤ ጥራት ምን ደረጃ ይሰጡታል?<br>Overall, taking everything into account, how would you rate the quality of care you received at this facility for this delivery?	1 እጅግ በጣም ጥሩ Excellent<br>2 በጣም ጥሩ Very good<br>3 ጥሩ Good<br>4 በቂ Fair

		5 ደካማ Poor
32	በአጠቃላይ በጤና ተቋሙ የተሰጥዎት አገልግሎት ምን ያህል አርክትዎታል?<br>Overall, how satisfied are you with the way health care services are provided in this facility?	1 በጣም ረክቻለሁ<br>Very satisfied<br>2 የተወሰነ ረክቻለሁ<br>Somewhat Satisfied<br>3 አልረካውምም ረክቻለውምም አልልም<br>Neither satisfied nor dissatisfied/neutral<br>4 የተወሰነ አልረካውም<br>Somewhat dissatisfied<br>5 በጣም አልረካውም<br>Very dissatisfied
D	የክብር እና የእንክብካቤ ማጣት ገጠመኞች Experience of Disrespect and Abuse	
	አሁን ስላለዎት ልምድ እጠይቅዎታለሁ፡፡ ለጥያቄዎች ትክክለኛ የሆነ እና ያልሆነ የሚባል መልስ የለም፡፡ የሚሰጡን ማንኛውም መረጃ ከእርስዎ እና ከልጆችዎ ስም ጋር የማይገናኝ እና እንደዚሁም እርስዎም ሆነ የቤተሰቦችዎ አባላት አሁንም ወደፊት ከጤና ተቋሙ የሚያገኙት አገልግሎት ላይ ተጽዕኖ የማያስከትል ነው፡፡ ለሚሰነዝርልዎት ጥያቄ ምኞት ካልተሰማዎች ምላሽ አለመስጠት ወይም ማለፍ ይችላሉ፡፡ Now I will ask about your experience. There is no right or wrong answers to these questions. Nothing you tell us will be linked to your name, your children's names, or the ability of you or your family members to access health care in the future. You can skip any question you are not comfortable answering, and you can stop the interview at any point.	
33	አሁን መጨረሻ ላይ በነበርዎት የወሊድ ጊዜ ከደረጃ 1-5 ባለው ልኬት መሠረት የእርስዎን ስብህና በሚጋፋ መልኩ ምቾትዎን የሚነሳ ግልጋሎት አግኝተዋል?<br>In your recent delivery, on a scale of 1 to 5, were you treated in a way that made you feel humiliated or disrespected?<br><br>ከ1-3 ያለውን ምላሽ ተመልክተው ተገቢውን መልስ ይስጡበት<br>[READ RESPONSE CATEGORIES 1 TO 3]	1.ክብሬ ተነክቷል<br>Humiliated disrespected<br>2. የተወሰነ ክብሬ ተነክቷል Somewhat humiliated/disrespected<br>3. በፍጹም ክብሬ አልተነካም<br>Not humiliated or disrespected at all
34	**በፈቃደኝነት ላይ ያልተመሰረተ የህክምና አሰጣጥ፡-** በዚህ ወሊድ ሳቢያ በጤና ተቋም በቆዩበት ጊዜ ያለእርስዎ ይሁንታ እና ፈቃድ የተሰጥዎት የህክምና አገልግሎት አለ?<br>Non-consented care: At any point during your stay for this delivery was any treatment done to you without your permission?	1 አዎ Yes<br>0 የለም - No→GOTO 36
35	**በፈቃደኝነት ላይ ያልተመሰረተ የህክምና አሰጣጥ፡-** ያለእርስዎ ፈቃድ የተደረገብዎት የህክምና ሂደቶች አሉ?<br>**Non-consented care:** What procedures were done without your permission?<br>(አያንቡት፣ የሚመለከትዎት ሁሉም ላይ ያክቡ ሌላም ካለ ይጥቀሱ) (DO NOT READ, Circle all that apply, prompt for any more)	1 የቱቦ መከተት Tubal ligation<br>2 የማህጸን ቀዶ ጥገና Hysterectomy<br>3 የሆድ መዳሰስ ህክምና<br>Abdominal palpation<br>4 የብልት ምርመራ<br>Vaginal examination<br>5 እስቲች Episiotomy<br>6 ጨቅላ ህጻን ከእናት መለየት<br>Newborn removed /separated<br>7 ሌላ Other<br>____________________
36	**ሚስጥራዊ ያልሆነ የህክምና አገልግሎት፡-** በህክምና አሰጣጡ ላይ ስለነበርዎት ግላዊነት እጠይቅዎታለሁ፡፡ ግላዊነት ማለት ከቤተሰብ አባላት ውጪ የሆኑ ግለሰቦች እርስዎ እንዲታይቦት ወይም እንዲሰማቦት የማይፈልጉትን ነገር ሌላ ወገን ተደራሽነት እንዳይኖረው መሆን ማለት ነው፡፡ በዚህ ወሊድ ወቅት በጤና ተቋሙ በቆዩበት ጊዜ ግላዊነቱን የሚጋፋ	1 አዎ Yes<br>2 የለም No→GOTO 38

	ክስተት ነበር?<br>**Non-confidential care:** Now I'd like to ask you about the privacy you had. Privacy relates to keeping out of view or hearing distance of others (non-family members) things that are normally not viewed or heard by others. At any point during your stay for this delivery were you treated in a way that violated your privacy?	
37	**ሚስጥራዊ ያልሆነ የህክምና አገልግሎት፡-** በትክክል የሆነው ምንድን ነው? (አያንቡት፣ የሚመለከትዎት ሁሉም ላይ ያክቡ ሌላም ካለ ይጥቀሱ)<br>**Non-confidential care**: What exactly happened? (DO NOT READ, Circle all that apply, prompt for any more)	1 በወሊድ ወይም ምርመራ ወቅት ያላግባብ ልብሴ ተገላልጧል፣<br>Uncovered during delivery or examination<br>2 በወሊድ ወይም ምርመራ ወቅት መሸፈኛ ወይም መጋረጃ አልተጠቀሙም፣<br>No screens blocking view during delivery or examination<br>3.የኤችአይቪ ሁኔታ የጤና ባለሙያ ላልሆነ ሶስተኛ ወገን ይፋ ተደርጓል፣<br>HIV status shown to others or discussed with non-health staff<br>4. ሌላ የጤና መረጃዬ ለሶስተኛ ወገን ወይም የጤና ባለሙያው አባል ላልሆኑት ወገኖች ይፋ ተደርጓል፣<br>Other health information shown to others or discussed with non-health staff<br>5.ስለእኔ ጉዳይ ሌሎች ወገኖች እየሰሙ ባለሙያው ተናግሯል፣<br>Discussed my issues when other clients are listening<br>6. ሌላ ካለ ይጥቀሱ Other ___________________
38	**ክብርን የሚጋፋ ድርጊት፡-** በእናቶች የወሊድ እና ጽንስ አገልግሎት ክፍል ሳሉ የክፍሉ ሠራተኛ የእርስዎን ክብር የሚነካ ነገር ሰርተዋል ወይም ፈጽመዋል፣ ሰድበዎታል፣ ስለራስዎ መጥፎ ስሜት እንዲሰማዎት የሚያደርግ ነገር አድርጓል?<br>**D&A:** During the time you were at the maternity care facility, did the staff say or do something to humiliate you, insult you, or make you feel bad about yourself?	1 አዎ Yes<br>2 የለም No →GOTO 42
39	**ክብርን የሚጋፋ ድርጊት፡-** በእናቶች ህክምና ክፍል ሳሉ በምጥ ወይም በወሊድ ወቅት የክፍሉ ሠራተኛ በሚሰጥዎት የህክምና አገልግሎት ዙሪያ ችግር አለብዎት?<br>**D&A:** Did you have a problem with the way the staff treated while you were at the maternity care facility for labor, birth?	1 አዎ Yes<br>2 የለም No
40	**ክብርን የሚጋፋ ድርጊት፡-** በትክክል የሆነው ምንድን ነው?<br>(አያንቡት፣ የሚመለከትዎት ሁሉም ላይ ያክቡ ሌላም ካለ እያሉ ይጠይቁ)<br>**D&A:** What exactly happened?<br>(DO NOT READ. CIRCLE ALL THAT APPLY.<br>PROMPT: "Any More?")	1 ጮህውብኛል Shouted<br>2 አመናጭቀውኛል Scolded<br>3 የህክምና አገልግሎት እንደሚከለክለኝ አስፈራርተውኛል<br>Threatened to withhold services

		4 ወደ ቀዶ ጥገና እናስገባሻለን ብለዉ አስፈራርተውኛል<br>Threatened with going to theatre<br>5 የስድብ ስም በመጠቀም ጠርተውኛል<br>Called by insulting name<br>6ስቀውብኛል ወይም የሹፈት ምልክት ተጠቅመዋል<br>laughed at or scorned<br>9 ሌላ Other<br>____________________
41	ከላይ የተጠቀሱት ክብርን የሚነካ ድርጊት ፈጽመዋል የሚሉት ሠራተኛ ማነው?<br>Which staff did the thing you mentioned above?	1.የህክምና አገልግሎት የሰጠኝ ባለሙያዎች<br>The health worker who attended me<br><br>2 የጽዳት ሠራተኞች<br>Janitors<br>3.የጥበቃ ሠራተኞች<br>Guards<br>4.ሌሎች ሠራተኞች<br>Other staff (explain)__________
42	ክብርን የሚጋፋ ድርጊት፡- በምጥ ወይም ወሊድ ወቅት የጤና ባለሙያው የአካል ጥቃት/በጥፊ መምታት ወይም ቁንጥጫ አድርሶብዎታል?D&A: During labor or delivery, were you physically hit /slapped/ pinched by the provider?	1 አዎ Yes<br>2 የለም No
43	ችላ መባል፡- ለወሊድ አገልግሎት በጤና ተቋሙ በቆዩበት ጊዜ ብቻዎትን ተጥለው (የጤና ባለሙያ ክትትል አጥተው) የህክምና ድጋፍ ቢያስፈልግዎትም ያላገኙበት ሁኔታ አለ? (አያንቡት፣ የሚመለከትዎት ሁሉም ላይ ያክቡ ሌላም ካለ ይጥቀሱ) Abandonment/ Neglect: At any point during your stay for this delivery were you left alone (unattended) by health providers when you needed care?	1 አዎ Yes<br>2 የለም No →GOTO 45
44	መጣል እና ችላ መባል፡- የትኛው የምጥ ወይም የወሊድ ደረጃ ላይ እያሉ ነው የህክምና ድጋፍ እና አገለግሎት እያስፈለግዎት ያላገኙት? Abandonment/ Neglect: At which point during your stay for this delivery were you left unattended?<br><br>(DO NOT READ. CIRCLE ALL THAT APPLY. PROMPT: "Any More?")	1 በምጥ ወቅት<br>While in labor<br>2 በወሊድ ወቅት<br>While delivering<br>3 የወሊድ የጎንዮሽ ጉዳት በታየብኝ ወቅት<br>While experiencing a complication<br>4 ከወሊድ በኋላ ከልጅ ጋር በተያያዘAfter delivery<br>Baby after delivery<br>8 ሌላ ካለ ይጥቀሱ Other<br>_____________________
45	ችላ መባል፡- በጤና ተቋሙ በቆዩበት ወቅት የህመም ማስታገሻ/ሌላ ህክምና እንዲሰጥዎት ጥያቄ አቅርበዋል?<br>Abandonment/ Neglect: At any point during your stay did you ASK for pain relief /other medication?	1 አዎ Yes<br>2 የለም No →GOTO 47

46	ችላ መባል፡- በጤና ተቋም በቆዩበት ጊዜ የህመም ማስታገሻ/ሌላ ህክምና ጠይቀው ችላ የተባሉበት ጊዜ አለ ብለው የሚገልጹት ካለ?Abandonment/ Neglect: At any point during your stay did you feel ignored regarding requests for pain relief /other medication?	1 አዎ Yes<br>2 የለም No
**E.**	የወደፊቱ ህይወት Looking Forward	
	አመሰግናለሁ፡፡ አሁን ወደፊት የጤና ተቋሙ ሊያደርግልዎት ስለሚገባው የህክምና አገለግሎት ያለዎትን እቅድ እጠይቅዎታለን፡፡<br>Thank you. Now I would like to ask you about your intentions or plans to choose health care facilities in the future.	
47	ተጨማሪ ልጆች መውለድ ይፈልጋሉ? Do you intend to have more children?	1 አዎ Yes<br>2 የለም No →GOTO 58
48	ቀጣይ የሚወልዱትን ልጅ የት መውለድ ይፈልጋሉ? Where would you like to deliver your next child?	1 በተመሳሳይ ጤና ተቋም<br>Same facility<br>2 በሌላ ጤና ተቋም<br>Another facility<br>3 በመኖሪያ ቤት<br>Your Home<br>9 ሌላOther (specify) ______________
49	በቀጣይ ጊዜ በምጥ ወይም በወሊድ ወቅት ድጋፍ የሚሰጥዎት ሌላ አጃቢ እንዲኖርዎት እፈልጋለሁ? Next time, would you like anyone to be with you during labor and delivery, for support?	1 አዎ Yes<br>2 የለም No →GOTO 51
50.	በቀጣይ ጊዜ በየትኛው መገኛ ወሊድ መስጠት ይፈልጋሉ?<br>Next time, at what location would you like to give birth?	1 በተመሳሳይ ጤና ተቋም<br>Same facility<br>2 በሌላ ጤና ተቋም<br>Another facility<br>3 በመኖሪያ ቤት<br>Your Home<br>9 ሌላOther (specify) ______________
51	ሌሎች እናቶች በዚህ ተቋም መጥተው እንዲወልዱ ምን ዓይነት ምክረ ሀሳብ ይሰጣሉ?<br>How likely are you to recommend this facility to other women for delivery?	1 ከፍተኛ እድል<br>Very likely<br>2 የተወሰነ እድል አለዉ<br>Somewhat likely<br>3 እድል ያለዉ አይመስለኝም<br>Somewhat unlikely<br>4 ምንም እድል የለውም Not at all likely
52	ወደፊት ቢያረግዙ በዚህ ጤና ተቋም የሚወልዱበት እድል ምን ይመስላል?<br>How likely are you to deliver in this facility again, if you were to become pregnant in the future?	1 ከፍተኛ እድል<br>Very likely<br>2 የተወሰነ እድል አለዉ<br>Somewhat likely<br>3 እድል ያለዉ አይመስለኝም<br>Somewhat unlikely<br>4 ምንም እድል የለውም Not at all likely

አመሰግናለሁ፡፡THANK YOU
የቃለ-መጠይቁ ያበቃበት ጊዜ፡- TIME INTERVIEW END: [RECORD TIME IN 24-HOUR [___|___:___|___]
