# Supplementary material for: Mistreatment of women in public health facilities of Ethiopia
Source: Reprod Health. 2019 Aug 27;16:130. doi: 10.1186/s12978-019-0781-y (PMC6712647; doi:10.1186/s12978-019-0781-y)
Supplement: Supplementary file 2 — RMC policy assessment tool. (PDF 151 kb) [file 12978_2019_781_MOESM2_ESM.pdf]

IRB #7168
Exempt Determination Date: 02May16
Version No.: 1

MCSP ETHIOPIA- MEASURING RESPECTFUL MATERNITY CARE (RMC) AMONG POSTPARTUM AND CHILD IMMUNIZATION CLIENTS

`

TOOL E: RMC RELATED POLICY AND PRACTICE ASSESSMENT TOOL

Name of HF__________________ HF ID______

Facility type:_____ 1. Health centre 2. Hospital

Region______________________ Zone ___________________Woreda________________

Select yes or no for the following practices and policies (Use observation when applicable)

	Y/N	Comment
**Policy related to RMC**		
Admission of family members/ person of choice to accompany women during labor/child birth		
A policy of allowing non harmful cultural rituals in the facility		
Requirement of informed consent for procedures		
Freedom of movement during labor (i.e., walking around)		
Allowing women choice of position for birth		
Policy of keeping the newborn with the mother immediately after the birth		
A policy of keeping mother and baby together throughout time in facility		
A policy that prohibit detention of women in facility for lack of payment		
Prevention of institutional violence against women and newborns		
**Infrastructure, materials, supplies**		
Adequacy of infrastructure to ensure privacy during labor and birth (privacy screens, curtains)		
Adequacy of infrastructure to ensure accommodation of companion during labor and birth		
Adequacy of infrastructure to ensure freedom of movement during labor		
Acquiring necessary materials or supplies (curtains, screens to ensure privacy)		
Availability of chairs for the birth companion; r and freedom to birth in vertical position		
Community involvement		
Feedback/anonymous reporting unprofessional behaviors		
Practice of facility tour (maternity open days) to pregnant women		
